# EXPLORING SOCIAL CONNECTEDNESS, PERSONALITY, HEALTH, AND LONELINESS AMONG OLDER ADULTS

**DOI:** 10.1093/geroni/igae098.4184

**Published:** 2024-12-31

**Authors:** Willencia Louis-Charles, Pallabi Bhowmick, Avinash Gupta, Wendy Rogers, George Mois

**Affiliations:** University of Illinois Urbana-Champaign, Champaign, Illinois, United States; University of Illinois Urbana-Champaign, Champaign, Illinois, United States; University of Illinois Urbana-Champaign, Champaign, Illinois, United States; University of Illinois Urbana-Champaign, Champaign, Illinois, United States; University of Georgia, Athens, Georgia, United States

## Abstract

As people age, changes in their social network may occur due to retirement, the loss of family and friends, and reduced social participation stemming from health issues, potentially leading to social isolation and feelings of loneliness. Approximately one in four older adults in the US alone are considered socially isolated. Being socially isolated is related to loneliness, but they are distinct constructions. Personality traits and perceptions of health may relate to loneliness perceptions. Personality traits are the enduring characteristics of a person and can predict how they will connect with their network. Older adults are also more likely to experience chronic conditions that impact their ability to socialize. As part of a larger study, we explored the relationships between social connectedness, personality, health, and loneliness for 21 older adults (61-80, M=69.0, SD=5.5) Participants completed the Ten-Item Personality Inventory and the Social Connectedness Scale, which integrates the Social Disconnectedness Scale and the Social Isolation Scale. A descriptive analysis was conducted to identify the participants’ personality types and self-reported social connectedness. Emotional stability was negatively correlated (r=-0.55) with perceived loneliness with participants having higher emotional stability also scoring high on the Social Connectedness Scale. No significant association was found between the other personality traits and loneliness. However, perceived health was positively linked to better social connection (r=0.51). These results provide insights into the potential role of personality and health for social connectedness that might help tailor potential interventions that could reduce loneliness among older adults, leading to a better quality of life.

